# Peripheral tissue perfusion in individuals with and without type 2 diabetes mellitus and its associations with type 2 diabetes: a cross-sectional study

**DOI:** 10.1590/1677-5449.202301062

**Published:** 2025-02-24

**Authors:** Valéria Cristina de Faria, Juliana Simões de Alencar Fernandes, Tulio Ericles de Oliveira Cunha, Guilherme de Azambuja Pussieldi, Danielle Aparecida Gomes Pereira

**Affiliations:** 1 Universidade Federal de Minas Gerais, Belo Horizonte, MG, Brasil.; 2 Universidade do Norte do Paraná, Betim, MG, Brasil.; 3 Universidade Federal de Viçosa, Florestal, MG, Brasil.

**Keywords:** leg, microcirculation, near-infrared spectroscopy, type 2 diabetes mellitus, membro inferior, microcirculação, espectroscopia de luz próxima ao infravermelho, diabetes mellitus tipo 2

## Abstract

**Background:**

Early recognition of peripheral tissue perfusion deficits can minimize secondary complications of peripheral arterial disease in individuals with diabetes.

**Objectives:**

To compare parameters of peripheral tissue perfusion in the leg at rest and during and after progressive effort between non-diabetics and individuals with type 2 diabetes and normal ankle brachial index values, as well as to evaluate the factors associated with peripheral tissue perfusion in the leg in individuals with type 2 diabetes during progressive effort.

**Methods:**

This cross-sectional study included 31 individuals with type 2 diabetes and 31 non-diabetics. Anthropometric measurements and physical activity levels were assessed in all individuals. Peripheral tissue perfusion was analyzed using near-infrared spectroscopy during an arterial occlusion maneuver and the Incremental Shuttle Walking Test.

**Results:**

During progressive effort, the tissue oxygen saturation level was lower in the type 2 diabetes group (type 2 diabetes, 58.74 [56.27–61.74] than the non-diabetic group, 62.15 [59.09–66.49]; p = 0.005). There were significant correlations between tissue oxygen saturation during progressive effort and physical activity level (p < 0.0001; r = -0.681), total body fat percentage (p = 0.001; r = 0.590), segmental body fat percentage (p < 0.0001; r = 0.616), total skeletal muscle mass (p < 0.0001; r = -0.628), and segmental skeletal muscle mass (p = 0.001; r = -0.592).

**Conclusions:**

Individuals with type 2 diabetes and normal ankle-brachial index values had worse tissue perfusion during progressive effort than non-diabetics, and there was an association between perfusion, physical activity level, and body composition in the type 2 diabetes group.

## Resumo

**Contexto:**

O reconhecimento precoce dos déficits de perfusão tecidual periférica pode minimizar as complicações secundárias da doença arterial periférica em indivíduos com diabetes.

**Objetivos:**

Comparar parâmetros de perfusão tecidual periférica do membro inferior em repouso e durante e após esforço progressivo entre indivíduos com diabetes tipo 2 (DM2) com índice tornozelo-braquial (ITB) normal e indivíduos sem diabetes. Além disso, avaliar os fatores associados à perfusão tecidual periférica do membro inferior em indivíduos com DM2 durante esforço progressivo.

**Métodos:**

Estudo transversal que avaliou 62 indivíduos: 31 com DM2 e 31 sem diagnóstico de diabetes (SDD). Em ambos os grupos, foram avaliadas medidas antropométricas e níveis de atividade física. A perfusão tecidual periférica foi analisada por espectroscopia de infravermelho próximo durante a manobra de oclusão arterial e o *Incremental Shuttle Walking Test*.

**Resultados:**

O nível de saturação tecidual de oxigênio (StO_2_) durante o esforço progressivo foi menor no grupo DM2 (DM2: 58,74 [56,27–61,74]; SDD: 62,15 [59,09–66,49]; p = 0,005). Houve correlações significativas entre o nível de StO_2_ durante o esforço progressivo e nível de atividade física (p < 0,0001; r = -0,681), percentual de gordura corporal total (p = 0,001; r = 0,590), percentual de gordura corporal segmentar (p < 0,0001; r = 0,616), massa muscular esquelética total (MME) (p < 0,0001; r = -0,628) e MME segmentar (p = 0,001; r = -0,592).

**Conclusões:**

Indivíduos com DM2 com ITB normal apresentaram pior perfusão tecidual durante o esforço progressivo em comparação aos indivíduos com SDD. Essa pior perfusão foi associada ao nível de atividade física e à composição corporal nos indivíduos com DM2.

## INTRODUCTION

The International Diabetes Federation estimates that by 2045 the worldwide prevalence of diabetes will increase by 46% in relation to the 2021 level.^1^ Among people with diabetes, peripheral arterial disease (PAD) is one of the main factors for morbidity and mortality.^2^ The main cause of PAD is atherosclerosis, which, although widespread, is more common in individuals with diabetes.^3,4^ Approximately 50% of people with diabetes also have PAD, a 5-10 times greater prevalence than among non-diabetics.^5^

Of note, more than 50% of PAD cases are asymptomatic and, hence, go undiagnosed and untreated. Although most cases do not lead to a serious disease, PAD could indicate impaired general vascular health and predict future lethal events.^6^ As an example, a Brazilian study of individuals with T2DM and no previous diagnosis of PAD identified PAD in more than a fifth of the sample. The authors highlighted hypertension, sedentary lifestyle, smoking, and overweight among the main risk factors.^7^ Moreover, PAD has a faster progression and worse outcomes in people in with diabetes.^8^

Thus, despite the clinical importance of detecting early PAD-related changes in individuals with diabetes, the diagnostic methods generally involve peripheral blood pressure measurement, which may not be reliable in diabetics due to the calcification of blood vessels.^8-10^ Thus, it is important to evaluate tissue oxygen saturation (StO_2_) in symptomatic and asymptomatic patients, since early recognition of perfusion deficits can reduce or prevent PAD complications in people with diabetes, such as ulcers and diabetic foot.^3^ Therefore, the growing interest in methods to assess tissue perfusion during rest and exertion in individuals with diabetes is justified.^4,11^

One such method is near-infrared spectroscopy (NIRS), which allows continuous non-invasive monitoring.^9^ However, in the clinical context, NIRS is not regularly used in diagnostic investigation of early metabolic and vascular changes in people with diabetes, especially type 2 diabetes,^9^ probably due to the required investment of money and time. Thus, determining whether there is an association between simple routine measurement and peripheral tissue perfusion could facilitate clinical practice and add information about tissue microcirculation in these patients. However, before considering these associations, it must be determined whether there is a difference in tissue perfusion pattern between non-diabetics and individuals with T2DM and normal ankle-brachial index (ABI) values. Related studies have either not evaluated individuals with T2DM,^12^ they did not assess the musculature most affected by PAD,^12,13^ or they did not perform measurements at rest.^14,15^

Thus, the present study’s objectives were: (1) to compare parameters of peripheral tissue perfusion of the leg at rest and during and after progressive effort between non-diabetics and individuals with T2DM with normal ABI values and (2) to evaluate the factors associated with peripheral tissue perfusion of the leg in individuals with T2DM during progressive effort.

## METHODS

This cross-sectional study was approved by the institutional research ethics committee (decision 2.395.601; certificate 78445417.1.0000.5149), as determined in Brazilian National Health Council resolution 466/12, in accordance with the Declaration of Helsinki. All participants provided informed consent prior to inclusion. This study has been reported according to STROBE criteria (https://www.equator-network.org/reporting-guidelines/strobe/).

### Sample

The non-diabetic sample was selected from a previous database of individuals^16^ recruited between August 2018 and July 2019 through advertisements in public spaces throughout the city. The T2DM sample was recruited in June and July 2019 by convenience according to the order of referral from the city’s Department of Health. A total of 62 volunteers participated in this study, 31 in the T2DM group and 31 in the non-diabetic group, matched by sex, age (± 3 years), and body mass index (± 3 kg/m^2^). All measurement conditions (e.g., environment, equipment, examiners, and collection routine) were identical for both groups.

**Figure 1:**
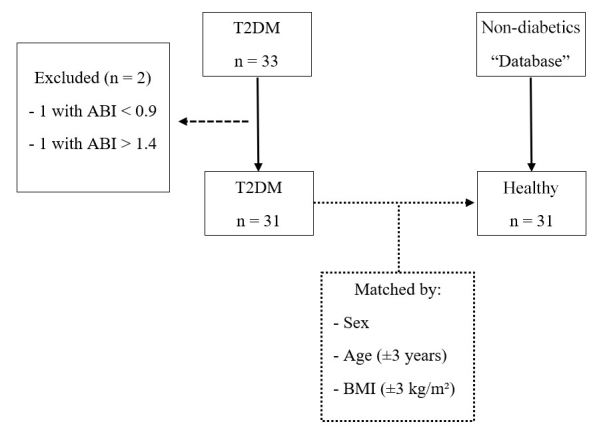
Sample selection flowchart. T2DM: type 2 diabetes; Non-diabetics; ABI: ankle-brachial index; BMI: body mass index.

The T2DM group included individuals of both sexes over 18 years of age who had been diagnosed with T2DM but had no musculoskeletal abnormalities or comorbidities that prevented physical effort and had medical clearance to participate in the study. Two individuals were excluded: one with a resting ABI value < 0.90 and one with a resting ABI > 1.40. The non-diabetic group included non-smokers of both sexes between 30 and 79 years of age who had not been diagnosed with diabetes, systemic arterial hypertension, kidney disease, angina symptoms, intermittent claudication, or any musculoskeletal changes that prevent physical exertion. The sample selection flowchart is shown in Figure 1.

The sample was calculated based on a previous correlation study,^9^ in which an average correlation coefficient (r) of 0.50 was obtained. With an alpha of 5% (Z_α_ = 1.960) and a power of 80% (Z_β_ = 0.842), the equation^17^ N=[(Z_α_+Z_β_)/C]^2^+3, where C=0.5*In[(1+r)/(1-r)], was applied, resulting in a sample size of 29 individuals per group.

### Measures and instruments

**Figure 2:**
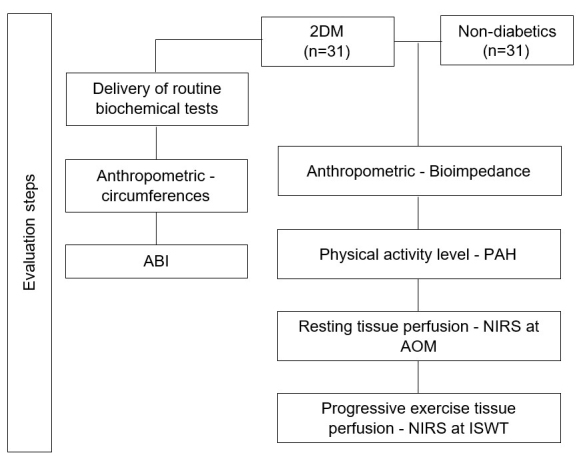
Flowchart of the assessment stages in the T2DM and Non-diabetic groups. T2DM: type 2 diabetes; Non-diabetic: individuals without diagnosis of diabetes; ABI: ankle-brachial index; AOM: arterial occlusion maneuver; HAP: Human Activity Profile; ISWT: Incremental Shuttle Walking Test; NIRS: near-infrared spectroscopy.

Figure 2 shows the assessment flowchart for both groups. After 10 min of rest in the supine position, participants in the T2DM group underwent an ABI evaluation according to guidelines in the literature.^18^ The lowest ABI value was used in the analysis.

As part of the anthropometric assessment, the waist, hip, and abdominal circumferences were measured with a tape measure (Cescorf, Porto Alegre, RS, Brazil) according to International Society for the Advancement of Kinanthropometry guidelines,^19^ and routine biochemical tests (fasting blood glucose, glycated hemoglobin, total cholesterol and cholesterol fraction, and triglyceride levels) were performed within 3 months of the evaluation.

Height was measured using a Balmak 111 scale stadiometer (Balmak Balanças, Santa Bárbara d’Oeste, SP, Brazil), and body mass and composition were assessed using an InBody R20 bioimpedance scale (InBody, Seoul, South Korea). Physical activity level was assessed with the Human Activity Profile activity-adjusted score.^20^

Tissue perfusion was measured with a NIRS device with a portable continuous wave system (PortaMon System, Artinis, Elst, the Netherlands), which uses two wavelengths of light (760 and 850 nm) to measure the concentrations of oxyhemoglobin and deoxyhemoglobin and calculate StO_2_. Before starting the NIRS procedure, the skinfold of the medial region of the sural triceps was measured with Cescorf adipometer calipers to assess the local adipose layer according to International Society for the Advancement of Kinanthropometry guidelines.^19^ The NIRS sensors were then positioned on the medial gastrocnemius muscle of the dominant leg (reported by the participant) and were fixed with plastic wrap and an elastic band.

### Near-infrared spectroscopy procedures

To assess StO_2_ values at rest, the participants remained resting for 10 min in the supine position and then underwent an arterial occlusion maneuver, which was performed using a cuff positioned on the distal third of the thigh and inflated to > 250 mmHg and maintained for 5–6 min.^21^ During and after this procedure, the following values were recorded: baseline StO_2_, the lowest StO_2_ value (minimal StO_2_), the deoxygenation rate (Tx-desox: the ratio of the difference between baseline StO_2_ and minimal StO_2_ and the time in seconds until minimal StO_2_ was reached), and reoxygenation rate (Tx-reox; ratio of the difference between baseline StO_2_ and minimal StO_2_ and the time until baseline StO_2_ was reached).

The participants were continuously monitored for 10 min after the NIRS assessment at rest and then performed the Incremental Shuttle Walking Test (ISWT)^22^ under continuous heart rate monitoring (Polar model FT1, Kempele, Finland). The results were recorded before the test, at the end of each stage, at the end of the test, and the end of the first minute of recovery. Blood pressure was recorded before the test, at the end of the test, and after recovery using a sphygmomanometer and stethoscope (P.A. Med, CBEMED, Itupeva, SP, Brazil). The test was interrupted when participants failed to stay on course (i.e., did not reach the cone) twice in a row^23^ or when the heart rate reached > 85% of the maximum [20], which was calculated using the formula: 208 − (0.73 × age in years).^24^ During and after the ISWT, NIRS was used to determine the following peripheral tissue perfusion variables: baseline StO_2_, minimal StO_2_, Tx-desox, Tx-reox, and resistance time (T-resist: time in seconds after the individual reached the lowest minimal StO_2_ until the end of the test). All NIRS data were collected, stored, viewed, and analyzed in OxySoft (Artinis Medical Systems, Elst, The Netherlands).

**Figure 3:**
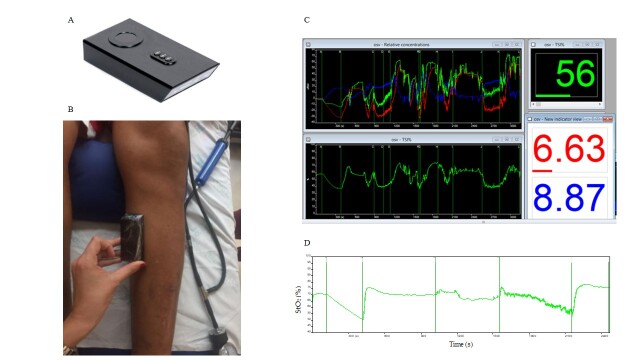
Illustration of the Near-Infrared Spectroscopy (NIRS) process: (A) the device; (B) the device applied to a patient; (C) software screen during measurement; (D) graph of a patient's StO_2_ during data collection.

Figure 3 illustrates the use of the NIRS device during data collection.

### Statistical analysis

Data normality was determined using the Shapiro-Wilk test. For the between-group comparison of parametric variables, the *t*-test for independent samples was performed, while for non-parametric variables, the Mann-Whitney U test was performed. For the association analysis, Spearman’s correlation coefficients were calculated. An alpha value of 5% was considered significant. The data were analyzed in IBM SPSS Statistics (IBM, Armonk, NY, USA).

## RESULTS

### Sample characteristics

Thirty-one participants were evaluated in each group. Both groups consisted of 17 women (55%) and 14 men (45%). Table 1 shows the general characteristics of the sample, with the only significant difference being physical activity level (Human Activity Profile) (T2DM, 76.16 [SD, 7.63]; non-diabetics, 82.16 [SD 9.11]; p = 0.007. Table 2 shows the specific data for the T2DM group.

**Table 1:** Demographic and clinical characteristics and physical activity level in the type 2 diabetes and non-diabetic groups.

Variables	T2DM (n = 31)	Non-diabetics (n = 31)	p
Age (years)	60.74 ± 10.18	60.23 ± 10.33	0.844
BMI (kg/m^2^)	30.50 ± 5.30	29.55 ± 4.75	0.458
Triceps skinfold (mm)	15.01 ± 9.36	19.07 ± 12.45	0.152
BFP total (%)	36.58 ± 9.67	35.97 ± 8.70	0.794
BFP segmental (%)	33.61 ± 9.43	33.46 ± 8.89	0.939
Total SMM (kg)	27.77 ± 6.93	26.95 ± 6.31	0.631
Segmental SMM (kg)	7.22 ± 1.84	7.11 ± 1.71	0.803
HAP	76.16 ± 7.63	82.16 ± 9.11	0.007*
ISWT (m)	435.16 ± 132.39	472.58 ± 136.19	0.277

Data are presented as mean ± standard deviation; T2DM: type 2 diabetes; BFP: body fat percentage; BMI: body mass index; HAP: adjusted Human Activity Profile score; ISWT: distance covered in the Incremental Shuttle Walking Test; SMM: skeletal muscle mass.

* p ≤ 0.05.

**Table 2:** Clinical characteristics of the type 2 diabetes group (n = 31).

Variables	T2DM
Time since diagnosis (years)	8.46 ± 5.72
Ankle-brachial index	1.11 ± 0.10
Waist-to-hip ratio	0.94 ± 0.83
Abdominal circumference (cm)	99.53 ± 20.08
Fasting glucose (mg/dL)	148.45 ± 61.65
Total cholesterol (mg/dL)	198.03 ± 32.00
HDL (mg/dL)	56.24 ± 14.37
LDL (mg/dL)	107.82 ± 36.79
Triglycerides (mg/dL)	146.07 ± 60.02
HbA1c (mmol/mol)	55 ± 10.16
HbA1c (%)	7.14 ± 1.32
Smoker (%)	2 (6.50)
Medications	
Beta-blockers (%)	8 (25.80)
Antihypertensives (%)	19 (61.30)
Hypoglycemics (%)	31 (100.00)
Insulin (%)	3 (9.70)

Data are presented as mean ± standard deviation and as absolute and relative frequencies. T2DM: type 2 diabetes; HDL: high-density lipoprotein; LDL: low-density lipoprotein; HbA1c: glycated hemoglobin.

### Incremental Shuttle Walking Test data

During the ISWT, the median heart rate variation (∆) (T2DM, 42 [33–65 bpm]; non-diabetics, 54 [41–71 bpm]; p = 0.049) and ∆SBP (T2DM, 20 [10–40 mmHg]; non-diabetics, 35 [20–50 mmHg]; p = 0.048) differed significantly between groups, while the ∆DBP (T2DM, 0 [-10–0 mmHg]; non-diabetics, 0 [-10–0 mmHg]; p = 0.562) was similar between the groups.

### Near-Infrared Spectroscopy data

Table 3 compares peripheral tissue perfusion values between the T2DM and non-diabetic groups; minimal StO_2_ during the ISWT was lower in the T2DM group (p = 0.005).

**Table 3:** Comparison between groups of peripheral tissue perfusion values.

	Variables	T2DM (n = 31)	Non-diabetics (n = 31)	p
**AOM**	Baseline StO_2_ (%)	65.01 (63.51–67.49)	64.77 (62.25–68.64)	0.540
	Minimal StO_2_ (%)	55.71 (50.27–59.58)	54.81 (45.60–59.81)	0.668
	Tx-desox (%/s)	0.03 (0.02–0.05)	0.34 (0.25–0.61)	0.218
	Tx-reox (%/s)	0.36 (0.23–0.41)	0.42 (0.17–0.55)	0.606
**ISWT**	Baseline StO_2_ (%)	66.62 (63.36–69.16)	68.72 (63.67–71.08)	0.113
	Minimal StO2 (%)	58.74 (56.27–61.74)	62.15 (59.09–66.49)	0.005*
	Tx-desox (%/s)	0.02 (0.01–0.03)	0.02 (0.01–0.03)	0.179
	Tx-reox (%/s)	0.08 (0.05–0.20)	0.11 (0.05–0.26)	0.762
	T-resist (s)	76.51 (51.06–96.20)	78.07 (46.24–125.83)	0.080

Data are presented as median and interquartile range. T2DM: type 2 diabetes; AOM: arterial occlusion maneuver; ISWT: Incremental Shuttle Walking Test; Baseline StO_2_: tissue oxygen saturation value immediately before the test; Minimal StO_2_: lowest tissue oxygen saturation value; Tx-desox: ratio of the variation between baseline StO_2_ and minimal StO_2_ on the time in seconds to reach minimal StO_2_; T-resist: time elapsed in seconds after the individual reached minimal StO_2_ until the end of the test.

* p ≤ 0.05.

**Figure 4:**
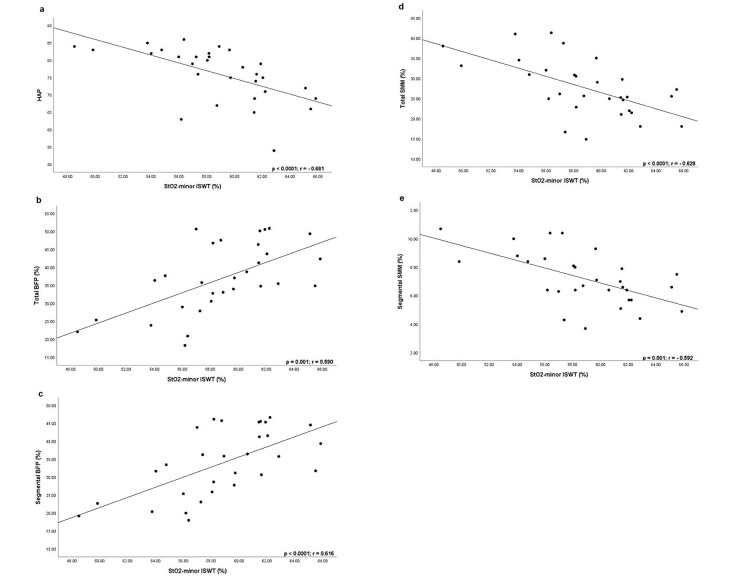
Correlation between minimal tissue oxygen saturation (StO_2_) value during the Incremental Shuttle Walking Test (ISWT) and (a) adjusted Human Activity Profile (HAP) score, (b) total body fat percentage (BFP), (c) segmental body fat percentage, (d) total skeletal muscle mass (SMM), and (e) segmental skeletal muscle mass.

Minimal StO_2_ was the only peripheral perfusion variable to significantly differ between groups during the ISWT, verifying its utility in association with simple routine measurements for individuals with T2DM. Figure 4 shows significant correlations between minimal StO_2_ during the ISWT and simple routine measurements. Other simple routine measurements included: time since diagnosis (p = 0.661; r = 0.084), smoking status (p = 0.080; r = -0.324), body mass index (p = 0.535; r = 0.118), waist-to-hip ratio (p = 0.215; r = -0.233), abdominal circumference (p = 0.789; r = 0.051), fasting blood glucose (p = 0.517; r = 0.128), glycated hemoglobin (p = 0.880; r = -0.029), total cholesterol (p=0.061; r= 0.352), high-density lipoprotein (p = 0.623; r = 0.097), and low-density lipoprotein (p = 0.351; r = 0.183).

## DISCUSSION

The main findings of this study were the significantly lower minimal StO_2_ during physical effort in the T2DM group than the non-diabetic group and significant associations of moderate magnitude between this variable and the adjusted Human Activity Profile score, total body fat percentage, segmental body fat percentage, total skeletal muscle mass, and segmental skeletal muscle mass.

In a recent study using NIRS, Manfredini et al.^25^ assessed individuals with PAD with and without diabetes, finding reports of lameness among those with diabetes, as well as a significantly lower degree of oxygenation in the medial gastrocnemius muscle compared to individuals with PAD alone. Considering these results and that muscle hypoxia can impair the performance of functional tasks and balance,^25^ the present study stands out in identifying that individuals with diabetes but no apparent PAD have worse peripheral tissue perfusion in the legs. This indicates that early intervention can be performed upon the appearance of functional limitations to mitigate complications.

Mohler et al.^14^ assessed blood volume during exercise using NIRS, reporting for the first time that blood volume expansion during exercise in individuals with T2DM without PAD was lower than in individuals with PAD alone. This indicates that impaired blood volume during exercise is a unique aspect of individuals with diabetes.^14^ This aligns with the lower minimal StO_2_ values found during progressive effort in our T2DM group, which reinforces the hypothesis that, even without PAD, individuals with diabetes have lower capillary volume expansion due to impaired vasodilation due to endothelial dysfunction,^26^ i.e., oxygen supply to active muscles is deficient.

Other studies have evaluated peripheral tissue perfusion behavior during exercise in individuals with T2DM using NIRS.^13,27^ However, these studies^13,27^ have found that muscle oxygenation is poorer in individuals with T2DM than non-diabetics. Since, according to Barker et al.,^28^ tissue perfusion values differ according to the body region, our study is important in that it investigates tissue perfusion of the muscle most affected by peripheral atherosclerotic phenomena, the sural triceps,^29^ mainly because it is assessed during a more functional activity, such as walking.

Manfredini et al.^30^ evaluated the metabolism of the medial gastrocnemius in individuals with PAD in comparison to healthy individuals, finding a compensatory response to significant heart rate increases in the PAD group, even though 21% of this group were using beta-blockers, thus maintaining the same volume of local blood in relation to non-diabetics. This increase in microvascular blood flow is expected due to the compensatory mechanism in response to impaired blood flow in the larger arterial vessels. However, individuals with diabetes cannot adjust their cardiac output,^31^ which was evident in our study, since the heart rate variation during effort was significantly lower in the T2DM group than the non-diabetic group. However, another possible explanation for this behavior is the use of beta-blockers, which reduced the heart rate^32^ by 25.80% in the T2DM group.

Another significant difference we found was the lower physical activity level in the T2DM group than the non-diabetic group. This was expected due to the decreased in physical activity among individuals with diabetes due to greater difficulty exercising.^33^ This may be because these individuals have fewer type I muscle fibers (mitochondrial dysfunction) or because they have lower microvascular response in the legs during exercise.^31^ Thus, in addition to central limitations, peripheral impairment is an important contributor to low physical fitness in individuals with diabetes.^11^ However, despite the significant difference in physical activity level, both groups were considered active (adjusted Human Activity Profile score > 74),^20^ which indicates that even among those who are physically active, peripheral tissue perfusion is lower in individuals with T2DM.

Thus, being classified in the same physical activity level (directly measured by distance covered in the ISWT) might explain the groups’ similar functional capacity. The fact that the T2DM group had no vascular alterations in the ABI could also explain this result. Otherwise, the common microvascular stiffness in individuals with symptomatic diabetes would limit oxygen delivery to the skeletal muscles, inducing early fatigue during exercise.^11^

Although StO_2_ is the most important direct parameter in clinical practice,^34^ assessment is not always possible. However, we highlight the inverse correlation between the minimal StO_2_ value during progressive effort and the adjusted Human Activity Profile score in the T2DM group. A higher physical activity level in individuals with diabetes may indicate adaptations to exercise, such as neovascularization and increased mitochondrial capacity.^35^ The fact that the training level interferes with the degree of deoxygenation in skeletal muscle could also explain this result.^36^

Another easy-to-apply measurement that was directly correlated with minimal StO_2_ during the ISWT was total and segmental body fat percentage, particularly the segmental percentage. This is probably because the thickness of the adipose tissue, especially that which is associated with NIRS positioning, can result in overestimated StO_2_ values_,_^37^ which can be extrapolated to the body fat percentage value. More significantly, the segmental body fat percentage only corresponds to the evaluated leg. Studies have shown that higher body fat is directly associated with lower muscle oxygen supply^38^ due to the lower metabolic rate and blood flow in adipose tissue; consequently, there are fewer changes in the NIRS signal.^36^

Finally, there was an inverse correlation between total and segmental skeletal muscle mass and minimal StO_2_ during the ISWT, which may have been due to the higher metabolic demand of skeletal muscle mass. This can be better explained in light of segmental skeletal muscle mass, since this correlation represents hyperemia in the active muscle during exercise, i.e., the greater the skeletal muscle mass, the greater the blood flow at the site,^39^ thus favoring tissue perfusion and resulting in a lower StO_2_ value during the ISWT.

ABI is the most common method of indirectly evaluating limb perfusion, although, like other peripheral blood pressure measurements, it may not be reliable in diabetics with calcified blood vessels.^40^ Consequently, in the present study we established ABI > 1.40 as an exclusion criterion for the T2DM group to control for this limitation and avoid false ABI results.

A previous study^16^ highlighted the clinical applicability of NIRS results in individuals with health conditions, since these conditions can affect the lower peripheral muscles, especially at the microcirculatory level and in asymptomatic individuals. Thus, when combined with other evidence, our results can facilitate early identification of changes in muscle perfusion in patients with T2DM and normal ABI, thus contributing to more assertive prevention measures.

One study limitation was the lack of blinding, which is common in studies with a cross-sectional design. Another limitation may be the sample size calculation, since it only considered the correlation analysis.

## CONCLUSIONS

Individuals with T2DM and normal ABI had lower tissue perfusion during progressive effort (minimal StO_2_ during the ISWT) than non-diabetics, and there was an association between perfusion, body composition, and physical activity level in the T2DM group. The behavior observed in individuals with T2DM and normal ABI values indicates early health changes, which deserves more thorough investigation.
